# Assessment of Outer and Middle Ear Pathologies in Lilongwe, Malawi

**DOI:** 10.3390/audiolres14030041

**Published:** 2024-05-30

**Authors:** Ruth Mtamo, Jenna Vallario, Ambuj Kumar, Jesse Casanova, Julia Toman

**Affiliations:** 1 African Bible College, Area 47, Lilongwe P.O. Box 1028, Malawi; jennavallario@gmail.com; 2 Department of Internal Medicine, Morsani College of Medicine, Tampa, FL 33612, USA; akumar6@usf.edu; 3 College of Public Health, University of South Florida, Tampa, FL 33612, USA; jcasanov@usf.edu; 4 Department of Otolaryngology, Head and Neck Surgery, Morsani College of Medicine, Tampa, FL 33612, USA; juliatoman@usf.edu

**Keywords:** conductive hearing loss, outer and middle ear pathologies, hearing health disparities, hearing health, low-income countries, ABCHCTC

## Abstract

Outer and middle ear pathologies are known to disproportionately affect low-income countries but data is limited. We aim to quantify the prevalence rate of patients presenting with middle/outer ear pathologies at ABC Hearing Clinic and Training Centre in Lilongwe, Malawi. Audiological consultations (adult and paediatric) from 2018–2020 were reviewed for outer and middle ear pathologies. Secondary outcomes included patient type (private vs. community) compared to otoscopy findings, tympanometry findings, need for follow up, and follow up compliance. Out of 1576 patients reviewed, the proportion of abnormal cases’ was 98.2%, with 41.4% being unilateral and 57.4% bilateral. Eighty-three percent presented with outer/middle ear pathologies. 68% of those presented with a pathology often associated with some degree of conductive hearing loss (occluding wax, perforation, discharge, Type B/Type C tympanogram). Average age was 29 + 0.527 years; 41.6% private and 58.2% community patients. Cerumen impaction was most common finding (51%). Higher rates of otoscopic abnormalities and type B tympanograms were noted in community vs. private patient (~40% vs. ~30%; ~70% vs. ~30%). Adherence to follow up was higher for community vs. private patients (29% vs. 17%); ~70% reported subjective improvement upon follow up. The majority required multiple interventions on follow up. Secondary follow up was recommended in 64.8%. A significant disease burden of outer and middle ear pathologies was identified. Further research is required to understand the disease burden and promote health policy.

## 1. Introduction

Hearing loss in one of the most common health issues on the rise in this era. With an estimate of 750 billion dollars as the annual cost, hearing loss is the fourth largest cause of disability globally [1]. Despite hearing loss being a global health issue, there are significant differences between high-income countries and low-income countries. In a study conducted by Stevens et al in 2013, prevalence of child and adult hearing impairment was substantially higher in middle- and low-income countries than in high income countries [2]. 

### 1.1. Malawi as a Low-Income Country

Located in the south-eastern part of Africa, Malawi is one of the poorest countries in the world [3]. As a low-income country, its gross national income per capita (GNI) is less than $1085 [4]. Three fourths of the Malawian population live on less than $1.90 per day [5]. Thus, access to health services including ear and hearing health services is a challenge to the majority of the population.

### 1.2. Prevalence of Hearing Loss in Malawi 

There is very little existing literature and only a few studies have been conducted on the prevalence of hearing loss in the Malawian context. In one study, Mulwafu found that 11.5% of four- to six-year-olds had bilateral hearing loss [6]. Additionally, only 29.5% of children who were identified with hearing loss were enrolled in school [6]. Hunt et al in 2017 conducted a cross sectional study which identified 24.5% of children aged four to six had a hearing loss in at least one ear and 12.5% had hearing loss bilaterally [7]. Assuming this can be generalized to the entire population of Malawi, around 390,504 children aged four to six have a hearing loss in at least one ear. Both of these studies however suffered from limitations in sample size and full description of type and severity of hearing loss.

Depending on which part of the ear has been affected, hearing loss can be classified into three types namely Conductive hearing Loss (CHL), Sensorineural Hearing Loss (SNHL) and Mixed Hearing Loss (MHL) [8]. SNHL is a result of disorders in the cochlea or auditory nerve and these structures are in the inner ear. This type of loss is generally irreversible because of damage to the hearing organ or auditory nerve itself. Conductive hearing loss (CHL) is caused by disorders of the outer and/or middle ear [8]. There is a specific interest in outer and middle ear pathologies including those that are often associated with conductive hearing loss. In many cases they are reversible via medication or in some cases via surgery if there has not been permanent damage [9,10]. 

However, the issue of outer and middle ear pathologies including those that are often associated with conductive hearing loss has not been quantified especially in a LIC like Malawi. Therefore, the aim of this study is to evaluate the proportion of abnormal cases of outer and middle ear conditions among patients presenting to ABC Hearing Clinic and Training Centre. By quantifying the extent of these conditions, we can conclude that an emphasis should be placed on increasing accessible ear-related services especially in underserved communities within Malawi. 

## 2. Materials and Methods

### 2.1. Study Design/Setting/Participants

A retrospective case chart review was performed for all consecutive patients seen at ABC Hearing Clinic and Training Centre (ABC HCTC) in Lilongwe, Malawi between 2018 and 2020. This clinic during this timeframe is the main auditory service clinic in the region. All patient charts were reviewed. Additional information was collected on patient age, patient type (community versus private), otoscopy findings, tympanometry results, interventions at initial appointment, recommended follow up and attendance of follow up. 

Patients of all ages were eligible for inclusion. All types of payment visits were eligible for inclusion, both community and private pay patients. Private pay patients include those who have a medical scheme (health insurance) and/or those who pay the full amount for services. Community patients are those who pay a subsidized amount for services based on income level. 

### 2.2. Outcomes

The primary outcome was incidence of outer or middle ear conditions including infection (purulent drainage), middle ear effusion, cerumen impaction, foreign body, fungus, other abnormality of the ear canal and middle ear structures. Secondary outcomes included patient type (private vs. community) compared to otoscopy findings, tympanometry findings, need for follow up, and whether recommended follow up was attended. Another outcome included variations of the findings in paediatric and adult patients. In addition to classifying each abnormal finding for the left and right ear, we also classified abnormal findings as unilateral and bilateral. When the abnormal finding was present in both the left and right ear, the abnormality was counted twice for the summary percentage and therefore may exceed one hundred percent.

### 2.3. Statistical Analysis

Participant characteristics were summarized using descriptive statistics where continuous variables were summarized as mean and standard deviations and categorical as percent/rate. The difference in categorical variables across compared groups were assessed using the fisher exact test/chi-square test. The statistical significance was set at 5% for all comparisons. All analyses were performed using the IBM SPSS statistical analysis package version 29.

## 3. Results

### 3.1. Patient and Procedure Characteristics

A total of 1576 patient consultation appointments were reviewed and one chart was excluded due to incomplete data for review. The average age was 29 (±0.527) years with 570 (36.2%) paediatric and 1005 (63.8%) adult patients. The paediatric population was classified as those under the age of 18 years. The breakdown of patient type showed 652 patients (41.6%) were private/self-pay patients and 917 patients (58.2%) were community or partial pay. 

Overall, 83.4% had some degree of middle and outer ear pathologies for the right ear and 83.8% in the left ear. These pathologies included infection (purulent drainage), middle ear effusion, cerumen impaction, foreign body, fungus or other abnormality of the ear canal and visible middle ear structures, type As, Ad, B and C tympanometry findings. About 68.8% of those had pathologies that are often associated with some degree of conductive hearing loss for the right ear and 68.6% for the left ear, respectively. These included occluding ear wax, perforation (with and without discharge), type B, and type C tympanometry findings. 

### 3.2. Outcomes

**Figure 1:**
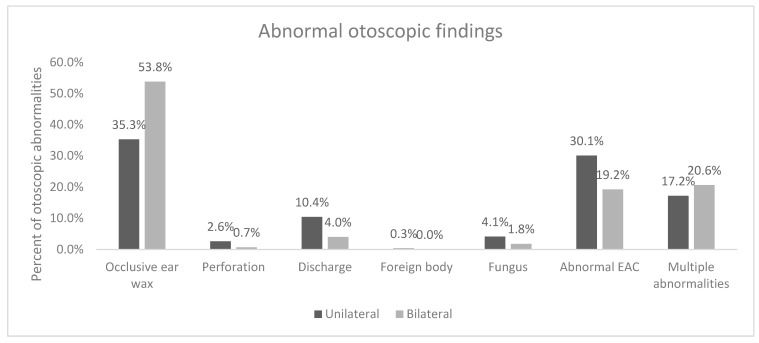
Breakdown of types of abnormalities noted on clinical otoscopic examination by unilateral or bilateral presentation.

The proportion of ear abnormalities (outer, middle and inner ear conditions) was 98.2%, with 41.4% being unilateral and 57.4% bilateral. There was a significant difference in unilateral versus bilateral abnormalities (*p* < 0.001). The most common abnormal unilateral otoscopic finding was occlusive ear wax noted in 35.3% of the patients, followed by abnormal EAC in 30.1%. The most common abnormal bilateral otoscopic finding was occlusive ear wax noted in 53.8% of the patients, followed by abnormal EAC in 19.2% (Figure 1). Overall, abnormal otoscopic findings were noted in 77.7% of left ear and 78.6% of right ears. 

**Figure 2:**
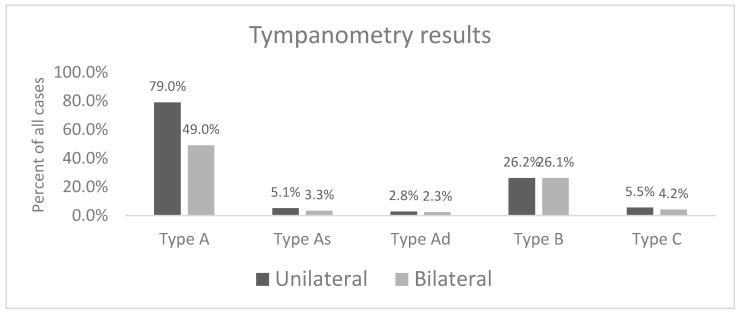
Tympanometry Results broken down by unilateral vs. bilateral findings. The percentage for each bar represents type of tympanometry finding and may overlap with other tympanometry finding due to bilateral presence of different type of abnormal tympanometry findings.

Results showed a Type A tympanogram was the most common finding in both ears (48.4% on the right and 47.1% on the left). The second most common tympanometry finding was a Type B tympanogram (18.8% for the right; 18.3% for the left). The proportion of Type A tympanogram was 79% for unilateral findings and 49% for bilateral findings (*p* < 0.001). The proportion of Type B tympanogram was 26.2% for unilateral abnormalities and 26.1% for bilateral abnormalities (*p* = 0.95). The proportion of Type C tympanogram was 5.5% for unilateral abnormalities and 4.2% for bilateral abnormalities (*p* = 0.23). The proportion of Type As tympanogram was 5.1% for unilateral abnormalities and 3.3% for bilateral abnormalities (*p* = 0.09). The proportion of Type Ad tympanogram was 2.8% for unilateral abnormalities and 2.3% for bilateral abnormalities (*p* = 0.62) (Figure 2). It should be noted tympanometry was performed post treatment for outer and middle ear conditions such as wax impaction, fungus, discharge, foreign bodies etc. Testing was not completed if an active infection and discharge was present.

Otoacoustic emissions (OAEs) were tested for the right and left ear. It should be noted that this test was only completed following treatment of middle and outer ear conditions. It was not performed in situations where testing was contra-indicated, such as patients with perforations, otitis media with effusion, etc. For the right ear, 51.5% of patients could not be tested, 43.8% of patients had “passed” findings and 4.8% “referred “findings. For the left ear, 51.6% could not be tested, 43.3% “passed” and 4.9% “referred”. Thirty seven percent of the patients with unilateral abnormalities could not be tested, 57% had “passed” findings and 6% “referred” versus 53% of patients with bilateral abnormalities could not be tested, 43% had “passed” finding, and 4% “referred” (*p* < 0.001). 

**Figure 3:**
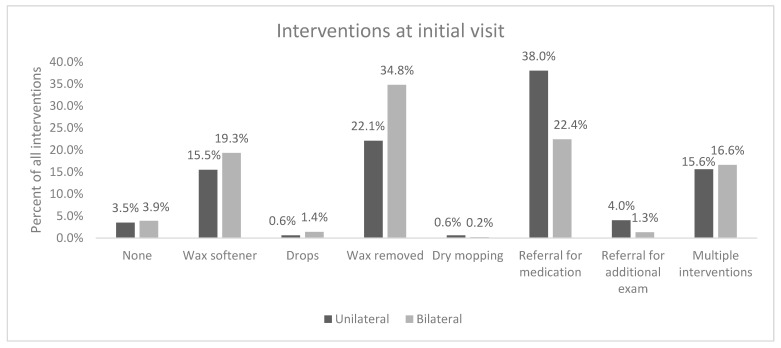
Interventions performed at time of initial appointment in unilateral vs. bilateral presentation.

Interventions at time of initial appointment showed that for the right ear, about 26.5% of patients had wax removed and another 15.5% had attempted wax removal but were ultimately given wax softener to use at home. Patients given wax softener were advised to return to clinic after one week for cerumen removal. Referral for medication, such as antibiotics and anti-fungal, was required for 21.9% of patients. For the left ear, intervention at time of initial appointment showed 25.6% of patients had wax removed, 15.5% were given wax softener, and 21.2% were referred for medication. There was a significant difference in initial interventions for unilateral and bilateral abnormalities (*p* < 0.001). The most common intervention at the time of the initial visit for unilateral abnormalities was a referral for medications (38%), followed by wax removal (22.1%) and wax softener (15.5%). The most common intervention at the time of the initial visit for bilateral abnormalities was wax removal (34.8%), followed by referral for medications (22.4%), wax softener administration (19.3%) and multiple, interventions (16.6%) (Figure 3).

With regard to follow up, 66% were recommended for follow up. However, only 46.2% of those patients attended the follow up. For those who did attend the follow up, ~70% reported subjective improvement (69.1% for the right ear, 71.1% for the left ear). Upon follow up, if an intervention was required, the majority required multiple interventions. Secondary follow up was recommended in 64.8% of cases. 

**Figure 4:**
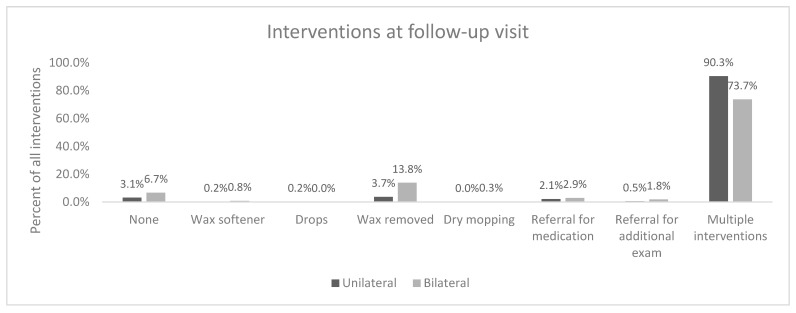
Interventions at follow up appointment.

There was a significant difference in follow up interventions for unilateral and bilateral abnormalities (*p* < 0.001). The most common intervention at the time of the follow up visit for unilateral and bilateral abnormalities was multiple interventions (90% for unilateral and 74% for bilateral (Figure 4).

Seventy-two percent of patients with unilateral abnormalities required follow-up versus 62.4% of patients with bilateral abnormalities (*p* < 0.001). Fifty-eight percent of patients with unilateral abnormalities attended the required follow-up versus 50% of patients with bilateral abnormalities (*p* = 0.017). On follow-up, 32% of patients with unilateral abnormalities reported improvement versus 26.4% of patients with bilateral abnormalities (*p* = 0.38). 

### 3.3. Subgroup Analysis 

#### Private Patients Versus Community Patients 

**Figure 5:**
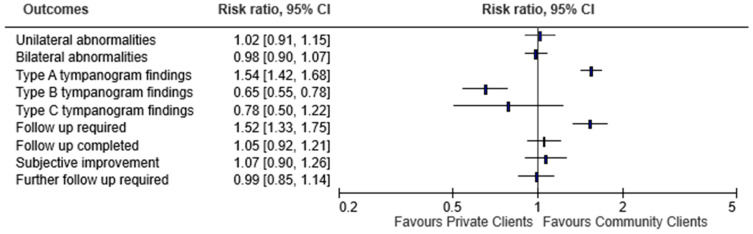
Distrubution of findings based on patient type (Private vs. community).

The percentage of disease pathology was evaluated by private versus community patients (Figure 5). When evaluated by patient type, abnormal otoscopy for the right ear was found in 32.3% of private patients and 46.1% of community patients (*p* = 0.576). For the left ear, abnormal otoscopic findings were noted in 32.4% private patients and 45.4% community patients (*p* = 0.951). There was no significant difference in the proportion of unilateral versus bilateral abnormalities for private versus community patients (*p* = 0.94). Forty-two percent of patients with unilateral abnormalities were private patients versus 41% with bilateral abnormalities. 

Type A and type B tympanometry findings were denoted by the following when evaluated by patient type. For type A, right ear was denoted by 24.5% of private patients and 24% of community patients. (*p* < 0.001). On the left side, type A tympanometry finding was indicated by 23.6% of private patients and 23.5% of community patients. (*p* < 0.001). There was a significant difference in type A tympanometry finding in private versus community patients for unilateral (83% for private vs. 76% for community; *p* = 0.04) and bilateral abnormalities (62% for private vs. 39% for community; *p* < 0.001). 

Type B tympanogram for the right ear was represented by 5.7% of private patients and 13.2% of community patients. (*p* < 0.001). The left ear indicated 5.9% of private patients and 12.4% of private patients for type B tympanometry finding. (*p* < 0.001). There was a significant difference in type B tympanometry finding in private versus community patients for unilateral (20.5% for private vs. 30.6% for community; *p* = 0.005) and bilateral abnormalities (19.7% for private vs. 30.6% for community; *p* < 0.001).

Type C tympanogram for the right ear was represented by 2.5% of private patients and 3.5% of community patients (*p* = 0.29). Type C tympanogram for the left ear was indicated in 2.5% of private patients and 3.4% of community patients (*p* = 0.37). There was no significant difference in type C tympanometry finding in private versus community patients for unilateral (4.4% for private vs. 6.4% for community; *p* = 0.30) and bilateral abnormalities (3.8% for private vs. 4.5% for community; *p* = 0.62). 

Intervention on initial appointment when evaluated by patient type showed 14.7% of private patients and 11.9% of community patients (*p* < 0.001) had wax removed for the right side. The left side was represented by 13.9% of private patients and 11.7% of community patients (*p* < 0.001). There was no significant difference in interventions on initial appointment for patients with unilateral abnormalities between private and community patients with most common intervention being referral for medication for private (32%) and community patients (*p* = 0.07). There was a significant difference in interventions on initial appointment for patients with bilateral abnormalities between private and community patients with most common intervention being wax removal for private (48%) and referral for medication (25.7%) along with wax removal (25.7%) for community patients (*p* < 0.001).

When evaluated by patient type, follow up required showed 23.9% of private patients and 42.2% of community patients (*p* < 0.001). There was a significant difference in requirement for follow-up for patients with unilateral (66% for private vs. 75% for community; *p* = 0.014) and bilateral abnormalities (52% for private vs. 70% for community; *p* < 0.001) between private and community patients.

Follow up attendance when evaluated by patient type showed 17.2% of private patients and 29% of community patients (*p* = 0.473). There was no significant difference in follow-up attendance in patients with unilateral (55% for private vs. 60% for community; *p* = 0.28) and bilateral abnormalities (50% for private vs. 50% for community; *p* = 1.00) between private and community patients.

Subjective improvement when evaluated by patient type denoted 27% of private patients and 42.1% of community patients (*p* = 0.104) for the right side while for the left side was 29.7% of private patients and 41.2% of community patients (*p* = 0.862). There was no significant difference in subjective improvement for patients with unilateral (72% for private vs. 66% for community; *p* = 0.52) and bilateral abnormalities (76% for private vs. 72% for community; *p* = 0.67) between private and community patients.

When evaluated by patient type, multiple intervention done on follow up indicated 32.8% of private patients and 44.1% of community patients (*p* = 0.476) for the left sided and 32% of private patients and 42.7% of community patients (*p* = 0.173) for the right side. There was no significant difference in requirement for multiple interventions for patients with unilateral (88% for private vs. 92% for community; *p* = 0.35) and bilateral abnormalities (78% for private vs. 71% for community; *p* = 0.33) between private and community patients.

Further follow up required when evaluated by patient type indicated 24.6% for private patients and 40.2% for community patients (*p* = 0.916). There was no significant difference in requirement for further follow-up for patients with unilateral (64% for private vs. 62% for community; *p* = 0.87) and bilateral abnormalities (65% for private vs. 67% for community; *p* = 0.77) between private and community patients (Figure 5).

### 3.4. Adult Patients versus Paediatric Patients 

**Figure 6:**
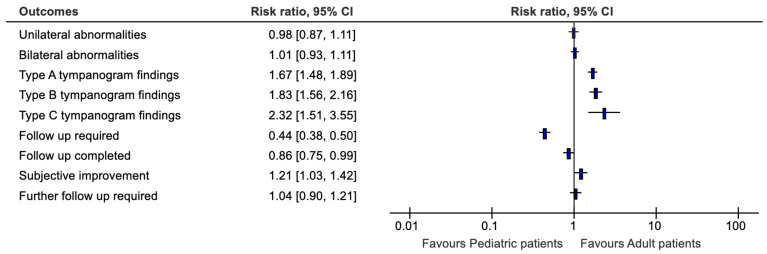
Distribution of findings based on age (Pediatric vs. Adult).

The disease pathologies were evaluated by adult versus paedatric patients (Figure 6). Evaluating otoscopic findings by age of patient gave abnormal otoscopic findings of 28.8% of paediatric patients and 49.8% of adult patients (*p* = 0.523) for the right side while the left side 27.4% of paediatric patients and 50.3% of adult patients (*p* = 0.147) for the left side. There was a significant difference in the proportion of unilateral versus bilateral abnormalities for pediatric versus adult patients (*p* = 0.013). Thirty-five percent of patients with unilateral abnormalities were pediatric patients versus 36% with bilateral abnormalities.

Tympanometry findings when evaluated by age of patient showed differences for type B of 13.6% of adult patients and 28.1.% of paediatric patients for the right side (*p* < 0.001). The left ear was denoted by 13.4% of adult patients and 26.8% of paediatric patients (*p* < 0.001). There was a significant difference in type A tympanometry finding in pediatric versus adult patients for unilateral (68% for pediatric vs. 85% for adults; *p* < 0.001) and bilateral abnormalities (33% for pediatric vs. 57% for adults; *p* < 0.001). There was a significant difference in type B tympanometry finding in pediatric versus adult patients for unilateral (34% for pediatric vs. 22% for adults; *p* = 0.001) and bilateral abnormalities (39% for pediatric vs. 19% for adults; *p* < 0.001). There was a significant difference in type C tympanometry finding in pediatric versus adult patients for unilateral (9.6% for pediatric vs. 3.3% for adults; *p* = 0.002) and bilateral abnormalities (6% for pediatric vs. 2% for adults; *p* = 0.038).

When evaluated by age of patient, otoacoustic emissions “refer” findings indicated 1.6% of paediatric patients and 3.2% of adult patients (*p* < 0.001) for the right side. The left side was represented by 1.8% of paediatric patients and 3.2% of adult patients (*p* < 0.001). There was no significant difference in otoacoustic emissions “refer”, “pass” or “could not test” findings in pediatric versus adult patients with unilateral (53% “pass” for pediatric vs. 59% for adults; *p* = 0.062) abnormalities. There was a significant difference in otoacoustic emissions “refer”, “pass” or “could not test” findings in pediatric versus adult patients with bilateral (31% “pass” for pediatric vs. 49% for adults; *p* < 0.001) abnormalities.

When further interventions at initial appointment was evaluated by age, it was indicated that 6.9% of paediatric patients and 19.6% of adult patients (*p* < 0.001) had ear wax removed on the right side. The left side was represented by 7.0% of paediatric patients and 18.5% of adult patients (*p* < 0.001). There was no significant difference in interventions on initial appointment for patients with unilateral abnormalities between pediatric and adult patients with most common intervention being referral for medication for pediatric (40%) and adult (37%) patients (*p* = 0.07). There was a significant difference in interventions on initial appointment for patients with bilateral abnormalities between pediatric and adult patients with most common intervention being referral for medication for pediatric (32%) and wax softener for adults (18%) patients (*p*< 0.001).

Follow up required when evaluated by age showed 26.3% of paediatric patients and 39.8% for adult patients (*p* < 0.001). There was no significant difference in requirement for follow-up for patients with unilateral abnormalities (73% for pediatric vs. 70% for adults; *p* = 0.468). There was a significant difference in requirement for follow-up for patients with bilateral abnormalities (73% for pediatric vs. 56% for adults; *p* < 0.001).

When evaluated by age follow up attended indicated 16.7% of paediatric patients and 29.4% of adult patients (*p* = 0.030). There was no significant difference in completion rates for follow-up for patients with unilateral (37% for pediatric vs. 45% for adults; *p* = 0.117) and bilateral abnormalities (46% for pediatric vs. 53% for adults; *p* = 0.145).

Subjective improvement on follow-up appointment when evaluated by age of patient gave 23.6% of paediatric patients and 45.3% of adult patients (*p*= 0.093) for the right side while for the left side it was 21.7% of paediatric patients and 49.4% of adult patients (*p* = 0.082). There was no significant difference in subjective improvement on follow-up for patients with unilateral (78% for pediatric vs. 66% for adults; *p* = 0.41) and bilateral abnormalities (83% for pediatric vs. 69% for adults; *p* = 0.128)

Differences in intervention on follow up were noted in multiple interventions when evaluated by age for 27.6% was of paediatric patient and 49.3% of adult patients (*p* = 0.120) for the left side while for the right side, 26.7% of paediatric patients and 47.9% of adult patients (*p* = 0.086). There was no significant difference in requirement for multiple intervention at follow-up for patients with unilateral (92% for pediatric vs. 90% for adults; *p* = 0.43) abnormalities. There was a significant difference in requirement for multiple interventions at follow-up for patients with bilateral abnormalities (71% for pediatric vs. 75% for adults; *p* = 0.029).

Further follow up was required by 24.1% of paediatric patients and 40.6% of adult patients (*p* = 0.596). There was no significant difference in follow-up requirements for patients with unilateral (70% for pediatric vs. 59% for adults; *p* = 0.24) and bilateral abnormalities (64% for pediatric vs. 68% for adults; *p* = 0.58) (Figure 6).

## 4. Discussion

The aim of this study was to quantify the proportion of outer and middle ear conditions at ABC Hearing Clinic and Training Centre in Lilongwe, Malawi. This is the largest evaluation of patients from a dedicated hearing clinic in this region with 1576 patient charts reviewed. The rate of patients with outer and middle ear pathology (83%) and then 68% of patients with pathologies that are often associated with conductive hearing loss indicates that middle and outer ear pathologies is a significant health burden to many Malawians. Compared to prevalence data from the Cape Town Metropolitan area showing ~13% rate of hearing loss in the population, the rates are extremely high [11]. 

Occluding ear wax was the most common abnormality found in this population. Cerumen impaction is a common outer ear pathology and, in some cases, it can be associated with conductive hearing loss. However, it is underrepresented in the literature. In developing countries, it has been reported that the most common ear etiology of hearing impairment is cerumen impaction with a prevalence range of 8.4% to 52.6% [12]. The rates found in this study at ~50% (bilateral) are on the higher end of the range reported in the literature. The second common finding was ear infections which were both common in adults and paediatric patients. This is attributed to poor living conditions and sanitation for the majority of the population. It is indicated by UNICEF that poor sanitation and hygiene are the key contributors to burden of disease and child survival. This costs the nation about US$57 Million annually or 1.1% of the national GPD as it causes increased health costs and yield in less productivity of the country [13]. Additionally, ear infections are known to disproportionately affect developing countries so the findings of this study are in keeping with the global trend [14,15].

There was a larger number of community patients seeking services at ABC HCTC than the private patients. An analysis of appointment attendance between community and private patients found community attendance at 58.2% and private attendance at 41.6%. There are definite financial barriers to accessing care and hearing healthcare resources in Malawi which may be extrapolated to explain the higher proportion of community patients compared to private paying patients. A survey conducted in 2019 of Malawians found that of 2958 participants, 39% of male headed homes and 59% of female headed homes had financial barriers to access health care services [16]. Further indications that there is limited access especially for community patients is the greater disease presence of abnormal otoscopy findings and higher rates of type B tympanograms among community patients. There is no other published data against which to compare this rate. It is possible the patients without means to access care routinely will present with greater severity of disease. 

Despite many patients being recommended for follow-up care, results indicated poor return rates. While two thirds of patients were recommended for follow up, less than half (46.2%) attended the follow up. This was true for both the private as well as community patients. This finding is similar to findings on the uptake rate on referrals in the southern part of Malawi [17]. Although these are different populations, it is evident that follow up attendance in most Malawian populations are low. 

Additionally, there was a significantly higher number of community patients who were recommended for follow up. One interpretation of this finding is that there is a higher disease burden which necessitated additional evaluation such as fluid in the middle ear space. Indeed, in the community patients, multiple interventions were required in a larger portion than the private patients although this was not significant. 

Interestingly, despite the presumed financial burdens, almost twice as many community patients attended the recommended follow up. In those patients who did follow up, nearly twice as many of the community patients noted subjective improvement compared to private patients although this was not significant. This may be related to the overall severity of disease which is potentially the reason for the higher portion of community patients recommended for a secondary follow up compared to the private client. 

The evaluation of paediatric compared to adult patients disclosed some unexpected results. The disease burden of some middle and outer ear pathologies such as ear infections, discharging ear, type B tympanogram as well as type C tympanogram had a higher proportion among adult population than the paediatric population with nearly twice as many adults with abnormal otoscopy findings compared to children. Typically some abnormalities that are often contributing to conductive pathologies especially related to eustachian tube dysfunction and otitis media are more common in the paediatric population [18]. Thus, the pattern of higher rates among the adult patients here indicates that there is a difference either in disease pathology or possibly patient population. One consideration is that it has been suggested that a draining ear in developing nations is considered a “normal” part of childhood, perhaps resulting in fewer children presenting for care [19]. In addition, in countries with advanced pediatric care, parents are educated on the importance of speech and language acquisition as it relates to normal hearing and auditory development. Given the lack of accessible hearing and ear-related care within Malawi, there is limited emphasis or priority placed on hearing health possibly resulting in an under-represented disease burden of transient pathologies within the pediatric population. 

With regard to follow up, adults had a higher rate of required follow up recommended. This is potentially due to the higher number of adult patients overall or greater burden of disease by the time patients present in adulthood. The attendance of the follow up was significantly less among the paediatric population compared to the adult patients. This is not surprising given that to bring a child requires additional transportation expense as well as time off work for the adult accompanying the child. Interestingly, adults noted nearly twice the rate of subjective improvement as the paediatric patients who did attend the follow up and adults were nearly twice as likely to require multiple interventions compared to paediatric patients. This finding however maybe due to limited ability of paediatric patients to communicate relative improvement depending upon age.

### 4.1. Significance of the Study

With appropriate diagnosis and timely intervention, conductive pathologies have the potential to be reversed. However, without the ability to quantify the disease burden, there is little motivation to address the issue which in many cases can be managed or alleviated with low-cost clinical interventions rather than expensive hearing amplification devices. In addition, if timely intervention is provided, it could prevent permanent damage to the outer/middle ear structures that would result in significant permanent conductive hearing loss. Therefore, providing low-cost clinical interventions is not only cost effective as it eliminates the need for expensive amplification, but it also saves money in the long run by reducing the incidence of permanent losses which will ultimately require life-long amplification and/or a surgical procedure. 

The extent of treatable conductive hearing loss is likely much broader than those who seek audiological services at ABC HCTC. By providing the proportion of outer and middle ear pathologies presenting specifically to ABC HCTC, this study will help various stakeholders, including the government, understand the importance of assisting those with treatable hearing loss.

### 4.2. Limitations to the Study

This study lacked some pieces of diagnostic tools to definitively identify hearing loss as an effect of these middle and outer ear pathologies. Instead, there was a usage of some abnormal otoscopic findings as well as tympanogram findings as a surrogate to associate the pathologies with some degree of hearing loss. Additionally, not only did the abnormal otoscopic and tympanogram findings serve as a surrogate to determine their association with hearing loss, but the study also had a shortfall in marking the severity of hearing loss. It should be noted that some of these outer/middle pathologies often range in severity of hearing loss. It is possible for the patient to experience hearing loss symptoms, yet air conduction pure tone audiometry results could remain within normal range. Often though, if air conduction thresholds are within normal range, there is a difference between air conduction and bone conduction thresholds, resulting in a conductive component (although within normal range) and associated reported audiological symptoms. 

Pure tone audiograms were not indicated for the majority of the participants. Out of 1576 participants, only 46 participants had pure tone audiometry performed at their consultation appointment. Therefore, a conductive loss pathology had to be presumed for some degree of conductive hearing loss but there is extremely limited data from the gold standard of pure tone audiometry to corroborate. 

Limitations on pure tone audiometry are likely related to a variety of factors mostly related to limitation of resources and finances. If clinically identified as a conductive pathology, PTA would not be undertaken until after treatment efforts had been undertaken. Following this, if the patient improved with treatment, it is a financial burden to the patient and the clinical resources to pursue PTA, so the number of patients receiving PTA with an initial conductive pathology are inherently limited due to these realities of practice limitations. Additionally, if the conductive component was treated prior to undertaking PTA, the conductive or mixed finding would be less likely to be captured once PTA is undertaken after treatment. These real-world factors thus limit the ability for gold standard PTA for hearing loss categorization.

## 5. Conclusions

Middle and outer ear pathologies are present and pervasive in low-income countries like Malawi. However, lack of their prevalence rates makes it difficult to quantify the significance of this matter. The significant disease burden of middle and outer ear pathologies including those that are often associated with some degree of conductive hearing loss described at the ABC Hearing Clinic and Training Centre speaks to the larger health burden which improved education and cost-effective intervention could work to combat. There is a significant need for additional studies to further evaluate the hearing loss disease burden in under resourced settings in order to better define public health policies and goals. 

## Data Availability

No new data were created or analyzed in this study. Data sharing is not applicable to this article.
